# Mendelian randomization supports causality between gut microbiota and chronic hepatitis B

**DOI:** 10.3389/fmicb.2023.1243811

**Published:** 2023-08-16

**Authors:** Quanzheng Zhang, Jinhua Zhou, Xiaoxiao Zhang, Rui Mao, Chuan Zhang

**Affiliations:** ^1^Department of Critical Care Medicine, The Third People’s Hospital of Chengdu, The Affiliated Hospital of Southwest Jiaotong University, Chengdu, Sichuan, China; ^2^West China Fourth Hospital, Sichuan University, Chengdu, China; ^3^Department of Dermatology, Xiangya Hospital, Central South University, Changsha, China

**Keywords:** gut microbiota, chronic hepatitis B, Mendelian randomization, genome-wide association study, linkage disequilibrium score regression

## Abstract

**Background:**

Observational studies have provided evidence of a close association between gut microbiota and the progression of chronic hepatitis B (CHB). However, establishing a causal relationship between gut microbiota and CHB remains a subject of investigation.

**Methods:**

Genome-wide association study (GWAS) summary data of gut microbiota came from the MiBioGen consortium, while the GWAS summary data of CHB came from the Medical Research Council Integrative Epidemiology Unit (IEU) Open GWAS project. Based on the maximum likelihood (ML), Mendelian randomization (MR)-Egger regression, inverse variance weighted (IVW), MR Pleiotropy RESidual Sum and Outlier (MR-PRESSO), and weighted-mode and weighted-median methods, we conducted a bidirectional, two-sample, MR analysis to explore the causal relationship between the gut microbiota and CHB. Additionally, we evaluated the genetic associations between individual gut microbes and CHB using the Linkage disequilibrium score regression (LDSC) program.

**Results:**

According to the IVW method estimates, genetically predicted class Alphaproteobacteria (odds ratio [OR] = 0.57; 95% confidence interval [CI], 0.34–0.96; false discovery rate [FDR] = 0.046), genus *Family XIII AD3011* group (OR = 0.60; 95% CI, 0.39–0.91; FDR = 0.026), genus *Prevotella 7* (OR = 0.73; 95% CI, 0.56–0.94; FDR = 0.022) exhibited a protective effect against CHB. On the other hand, family Family XIII (OR = 1.79; 95% CI, 1.03–3.12; FDR = 0.061), genus *Eggerthella* group (OR = 1.34; 95% CI, 1.04–1.74; FDR = 0.043), genus *Eubacterium ventriosum* group (OR = 1.59; 95% CI, 1.01–2.51; FDR = 0.056), genus *Holdemania* (OR = 1.35; 95% CI, 1.00–1.82; FDR = 0.049), and genus *Ruminococcus gauvreauii* group (OR = 1.69; 95% CI, 1.10–2.61; FDR = 0.076) were associated with an increased risk of CHB. The results from LDSC also indicated a significant genetic correlation between most of the aforementioned gut microbiota and CHB. Our reverse MR analysis demonstrated no causal relationship between genetically predicted CHB and gut microbiota, and we observed no significant horizontal pleiotropy or heterogeneity of instrumental variables (IVs).

**Conclusion:**

In this study, we identified three types of gut microbiota with a protective effect on CHB and five types with an adverse impact on CHB. We postulate that this information will facilitate the clinical prevention and treatment of CHB through fecal microbiota transplantation.

## Introduction

Nearly two billion people worldwide are affected by hepatitis B virus (HBV) infection, with 10–20% of them eventually developing decompensated liver cirrhosis or liver cancer, posing significant health risks (Trépo et al., 2014). The persistent nature of chronic hepatitis B (CHB) stems from a complex interplay between host immunity, the virus itself, environmental factors, and other variables, with impaired host immune function being a primary factor.

The gut microbiota refers to the complex community of microorganisms, including bacteria, viruses, fungi, and archaea, that reside in the digestive tracts of humans. This intricate ecosystem is comprised of trillions of microbes, with thousands of different species cohabitating and interacting within the host. The human gut microbiome is not only involved in essential metabolic processes but also plays a crucial role in immune regulation and maintenance of gut integrity (Chen et al., 2021; Ahmad et al., 2022; Rasheed et al., 2022). Current research suggests a potential link between gut microbiota and host immune function. Gut microbiota can directly or indirectly influence host immunity through the gut-liver axis (Compare et al., 2012; Milosevic et al., 2019). Under normal conditions, the interaction between gut microbiota and Toll-like receptors (TLR) on intestinal epithelial cells and immune cells helps maintain immune system homeostasis. In addition, gut microbiota maintains a dynamic balance with the liver by regulating metabolism and immune response and mediates immune and inflammatory responses by participating in bile acid metabolism (Yiu et al., 2017; Wang et al., 2020). Imbalances in gut microbiota may occur at all stages of CHB and may contribute to the chronicity of infection and the progression of the disease. Conversely, by regulating gut microbes, it may provide new avenues for the treatment of hepatitis B. Some studies are exploring how to use probiotics, prebiotics and other methods to improve the clinical outcome of hepatitis (Chen et al., 2011; Khoruts and Sadowsky, 2016; Morrison and Preston, 2016; Plaza-Diaz et al., 2019).

However, the causal relationship between gut microbiota and CHB remains uncertain. The comprehensive analysis of 86 independent studies by Sun et al. (2021). revealed that the changes in intestinal microbial metabolites abrogated the intestinal microbiota-bile acid-host axis, influencing the progression of CHB. Yet, several cross-sectional observations studies suggest that the occurrence and development of CHB can lead to alterations in gut microbiota (Chen et al., 2020; Zeng et al., 2020; Joo et al., 2021; Shen et al., 2023). Most earlier analyses were designed as case–control studies, making it challenging to ascertain exposure times and definitive outcomes. Additionally, in observational studies, the association between the gut microbiota and CHB is easily affected by confounding factors such as dietary patterns, environment, age, and lifestyle; and it is challenging to control these factors via observational studies effectively (Rinninella et al., 2019). These conditions limit the inference of a causal relationship between the gut microbiota and CHB.

Research design with MR follows the Mendelian inheritance law where “parental alleles are randomly assigned to offspring.” If genotype determines phenotype, the genotype is associated with a particular disease through the phenotype. Thus the association between CHB and gut microbiota can be inferred using the genotype as an instrumental variable. As an extension of the MR approach, bidirectional MR can be used to determine the direction of causality between two related phenotypes.

In the present study, we conducted a bidirectional, two-sample MR analysis adopting summary statistics of large GWAS from the MiBioGen consortium and the IEU Open GWAS project to assess the causal association between gut microbiota and CHB.

## Methods

### Data sources

We obtained the genetic variation in gut microbiotas from the largest genome-wide meta-analysis of gut microbiota to date conducted by the MiBioGen consortium (Kurilshikov et al., 2021; MiBioGen consortium, 2021). This study comprised 18,340 individuals from 24 cohorts (including the United States, Canada, Israel, South Korea, Germany, Denmark, the Netherlands, Belgium, Sweden, Finland, and the United Kingdom), targeting the variable regions V4, V3-V4, and V1-V2 of the 16S rRNA gene to analyse the microbial composition and classify the specific microbes using direct classification. We analysed microbiota quantitative trait loci (mbQTL) of microflora to identify the host genetic variation of genetic loci related to the abundance level of bacterial taxa in gut microflora. The genus was the lowest taxonomic level described in our study, and 211 taxa (131 genera, 35 families, 20 orders, 16 classes, and nine phyla) with an average abundance of more than 1% were then identified. The GWAS summary data (UK biobank, FinnGen consortium, and Biobank Japan) for CHB came from the data released by Sakaue et al. (2021), and these were then sorted and stored by the GWAS catalog (GWAS ID: GCST90018804, Trait name: Chronic hepatitis B) (IEU OpenGWAS project, 2021). The GWAS data included 19,082,360 single-nucleotide polymorphisms (SNPs) and 523,707 samples, including 2,379 in the case group and 521,328 in the control group. Age, sex, top 10 principal components, and genotyping batches were corrected during analysis (Sakaue et al., 2021).

### Instrumental variables

We used the following four criteria to select instrumental variables. First, the SNP-phenotype association level was required to reach the locus-wide significance threshold (*p* < 1 × 10^−8^). Unfortunately, only a small number of intestinal microbiota were selected as IV after we selected SNP. In order to explore the relationship between CHB and intestinal microflora to obtain more comprehensive results, we used the second threshold to identify a lower level of SNP- significance than the locus (*p* < 1 × 10^−5^) and selected them as the second IV set to find more potential causal associations. Second, SNPs with secondary (minor) allelic frequencies (MAFs) ≤ 0.01 were removed. Third, we only retained the lowest *p*-value among the SNPs with an *r*^2^ < 0.001 (with a clumping window size = 10,000 kb). When performing LD clumping on SNP data, we used EUR as a reference panel. Finally, we used the allelic frequency information to infer the forward strand alleles when palindromic SNPs were uncovered.

### Statistical analysis

**Figure 1:**
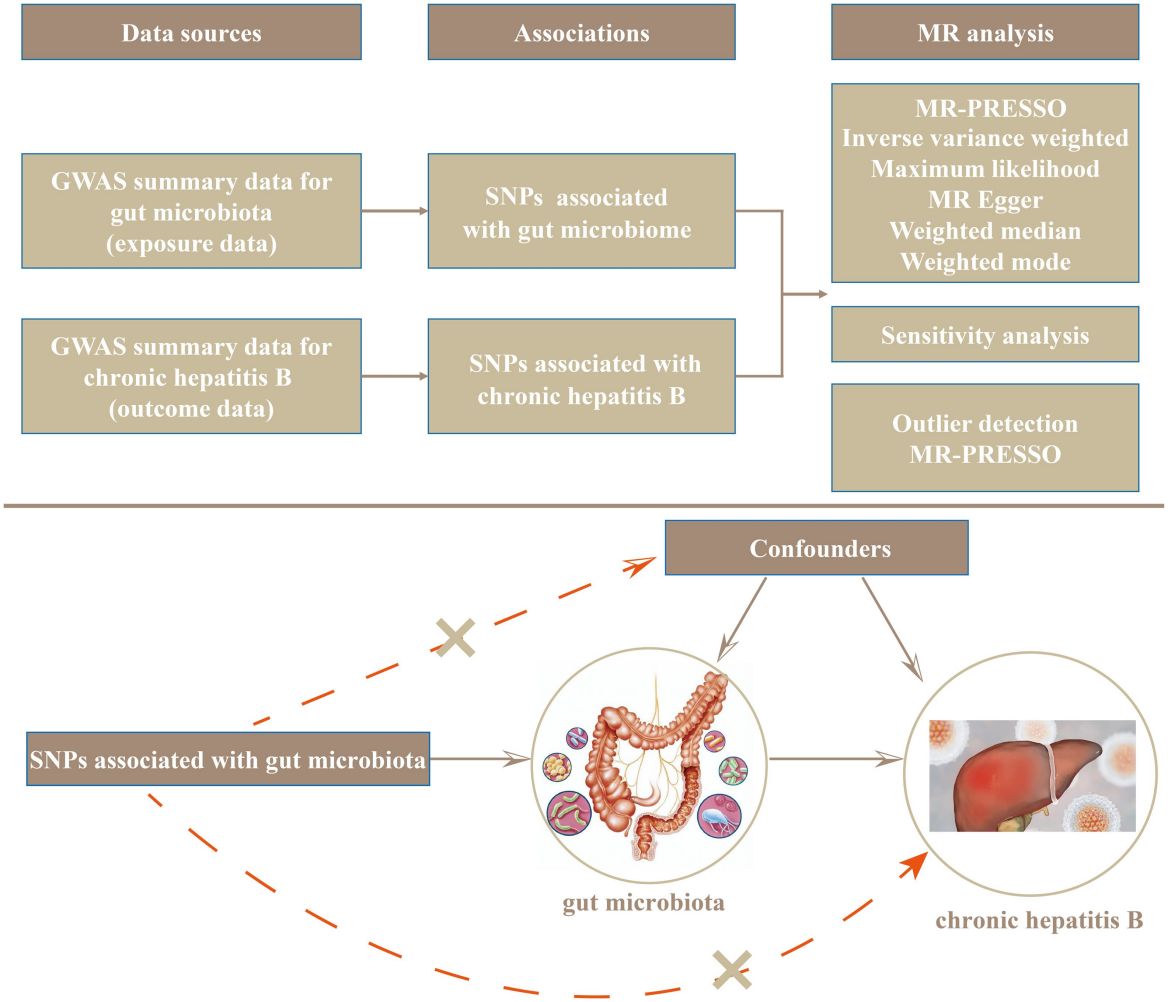
The design and workflow of the entire study.

The design and flow of the entire study are presented in Figure 1. In the present study, we evaluated the causal relationship between gut microflora and CHB using various methods, including maximum likelihood (ML), inverse variance weighted (IVW), MR Pleiotropy RESidual Sum and Outlier (MR-PRESSO), weighted-mode, MR-Egger regression, and weighted-median methods. IVW is characterized by ignoring the existence of an intercept term in regression and using the reciprocal of the outcome variance (se^2^) as the weight used for fitting. In the absence of heterogeneity and horizontal pleiotropy, IVWs constitute the most reliable estimates (Burgess et al., 2016). The ML method is similar to the IVW method, and under the assumption that there was no heterogeneity and that horizontal multiplicity was established, the evaluation result for ML showed almost no deviation, and its standard error was less than the IVW (Pierce and Burgess, 2013). MR-Egger regression is based on the assumption that instrument strength is independent of direct effect (InSIDE), which makes it possible to evaluate the existence of poly-efficacity with intercept terms. An intercept term of zero indicated that horizontal pleiotropy did not exist, and the MR-Egger regression result was consistent with IVW (Bowden et al., 2015). In addition, MR-Egger regression was also used to ascertain whether or not there was horizontal poly-tropism. When more than 50% of the instrumental variables are invalid, the weighted-median method can estimate causality more correctly. If the InSIDE hypothesis is violated, the weighted-model estimation method exhibits greater detection ability with regard to causal effect, less deviation, and a lower class-I error rate than with the MR-Egger regression (Hartwig et al., 2017). In addition, significant outliers were detected using MR-PRESSO (Verbanck et al., 2018) tests and MR-Egger regression, and horizontal pleiotropic effects were corrected by removing outliers. The global test also evaluates whether horizontal pleiotropy among all instruments is present (Verbanck et al., 2018). In addition, we further examined the heterogeneity among all SNPs using Cochran’s *Q*-test statistics. We identified potentially heterogeneous SNPs by conducting a “leave-one-out” analysis and excluding each instrumental SNP in turn. Finally, we also performed reverse MR analysis of CHB and gut microflora. The methods and settings we adopted were consistent with those of forward MR.

The F statistic was calculated using the formula $F=\left(\frac{R^{2}}{1-R^{2}}\times \frac{\left(n-k-1\right)}{k}\right)$ , where *R*^2^ was the proportion of the trait variance explained by the SNP, *k* was the number of IVs, and *N* was the sample size of the GWAS regarding the SNP for the particular trait; this was employed to quantify the strength of the instrument and a value >10 was considered sufficient. We used the TwoSampleMR R package to calculate the coefficient of determination (*R*^2^) regarding exposure to genetic variants. The *R*^2^ value was estimated using the formula $R^{2}=2\times \mathrm{EAF}\times \left(1-\mathrm{EAF}\right)\times {\left(\beta \right)}^{2}$ , where EAF was the effect allele frequency (EAF) of the SNP, SD was the standard deviation, and *β* was the estimated effect size of the SNP on the trait. Based on the online MR-power calculation tool^1^ (Burgess, 2014), we calculated the power of the MR estimates. Furthermore, we calculated the genetic association between each gut microbe and CHB using the LDSC program.

False discovery rate (FDR) correction was conducted by applying the fdrtools procedure, with a false discovery rate of *q*-value < 0.1 (Strimmer, 2008). All analyses were performed using R (version 4.2.2), and MR analysis was based on TwoSampleMR (Hemani et al., 2017) (version 0.5.6), meta (version 6.2.1) (Balduzzi et al., 2019), and MR-PRESSO (version 1.0) packages. The heatmap was drawn with the ImageGP tool (Chen et al., 2022).

## Results

### SNP selection

Based on the screening criteria of the IVs, we initially identified 1, 1, 3, 6, and 19 SNPs associated with gut microbiota at the phylum, class, order, family, and genus levels, respectively, at a significance level of *p* < 5 × 10^−8^. Details of each SNP, including specific chromosome, EAF value, and *R*^2^, are provided in Supplementary Table S1. However, the number of IVs obtained was not sufficient for a robust MR analysis. Therefore, under the screening of another threshold *p* < 1 × 10^−5^, we get 125, 224, 280, 503, and 1,667 SNPs associated with gut microbiota at the phylum, class, order, family, and genus levels. The details of SNP in the exposure and four outcome variables are shown in Supplementary Table S2. After excluding duplicated SNPs across different microorganisms, we got 21 SNP at a threshold of p < 5 × 10^−8^ and 1,676 SNP at a threshold of *p* < 1 × 10^−5^.

### Causal effects of gut microbiota on the development of CHB

Based on the IVs set filtered by a threshold of *p* < 5 × 10^−8^, We used gut microbiota as exposure and CHB as outcomes for MR analysis. However, due to the lack of IVs, the results of MR analysis can not be obtained. Therefore, we use the IVs filtered by the threshold of *p* < 1 × 10^−5^ to perform the following MR analysis (Kurilshikov et al., 2021; Li et al., 2022; Long et al., 2023).

**Figure 2:**
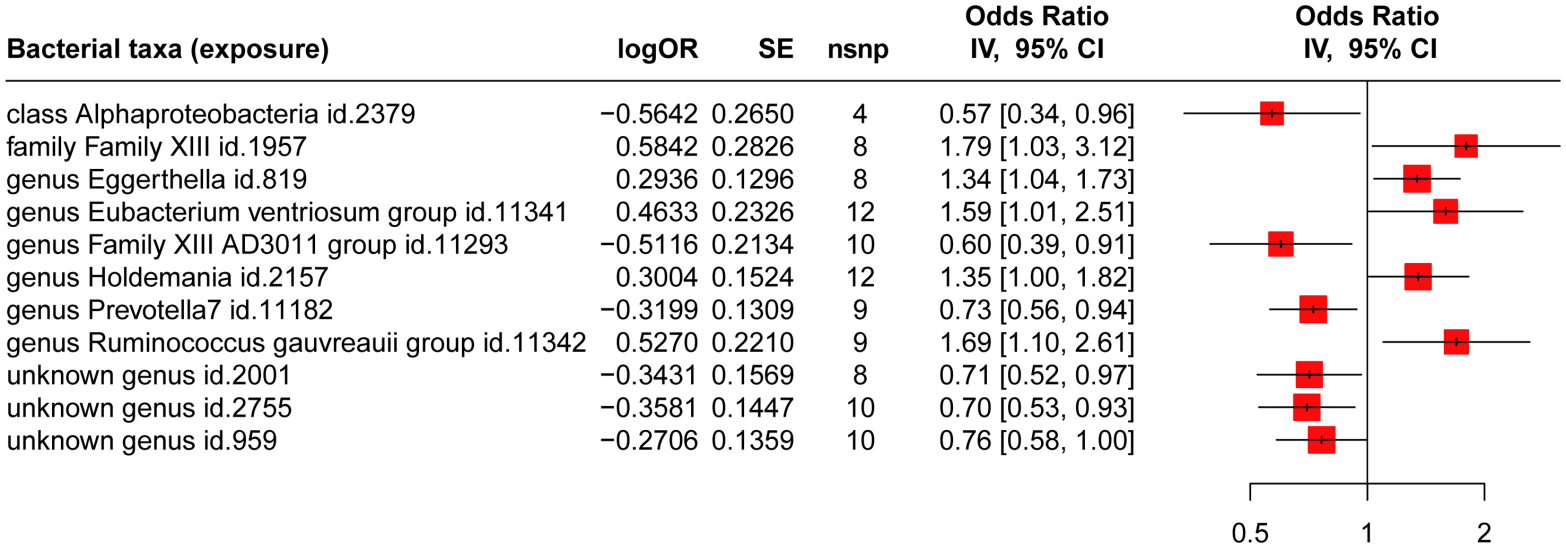
MR analysis of the causal relationship between genetically predicted gut microbiotas and CHB.

Four gut microbiotas were excluded because the available IVs were less than 3. The causal effects of the remaining 207 gut microbiotas on CHB are shown in Supplementary Table S3. Figure 2 illustrates 11 gut microbiotas that exert significant causal effects on CHB. Briefly, the estimate of IVW suggested that genetically predicted class Alphaproteobacteria (OR = 0.57; 95% CI, 0.34–0.96; *p* = 0.033; FDR = 0.046), genus *Family XIII AD3011* group (OR = 0.60; 95% CI, 0.39–0.91; *p* = 0.017; FDR = 0.026), genus *Prevotella 7* (OR = 0.73; 95% CI, 0.56–0.94; *p* = 0.015; FDR = 0.022), unknown genus id.2001 (OR = 0.71; 95% CI, 0.52–0.97; *p* = 0.010; FDR = 0.029), unknown genus id.2755 (OR = 0.70; 95% CI, 0.53–0.93; *p* = 0.013; FDR = 0.023), and unknown genus id.959 (OR = 0.76; 95% CI, 0.58–0.99; *p* = 0.047; FDR = 0.065) all exhibited a protective causal relationship with CHB. Conversely, genetically predicted family Family XIII (OR = 1.79; 95% CI, 1.03–3.12; *p* = 0.039; FDR = 0.061), genus *Eggerthella* (OR = 1.34; 95% CI, 1.04–1.74; *p* = 0.023; FDR = 0.043), genus *Eubacterium ventriosum* group (OR = 1.59; 95% CI, 1.01–2.51; *p* = 0.046; FDR = 0.056), genus *Holdemania* (OR = 1.35; 95% CI, 1.00–1.82; *p* = 0.040; FDR = 0.049), and genus *Ruminococcus gauvreauii* group (OR = 1.69; 95% CI, 1.10–2.61; p = 0.017; FDR = 0.076) exhibited a risk effect on CHB.

**Figure 3:**
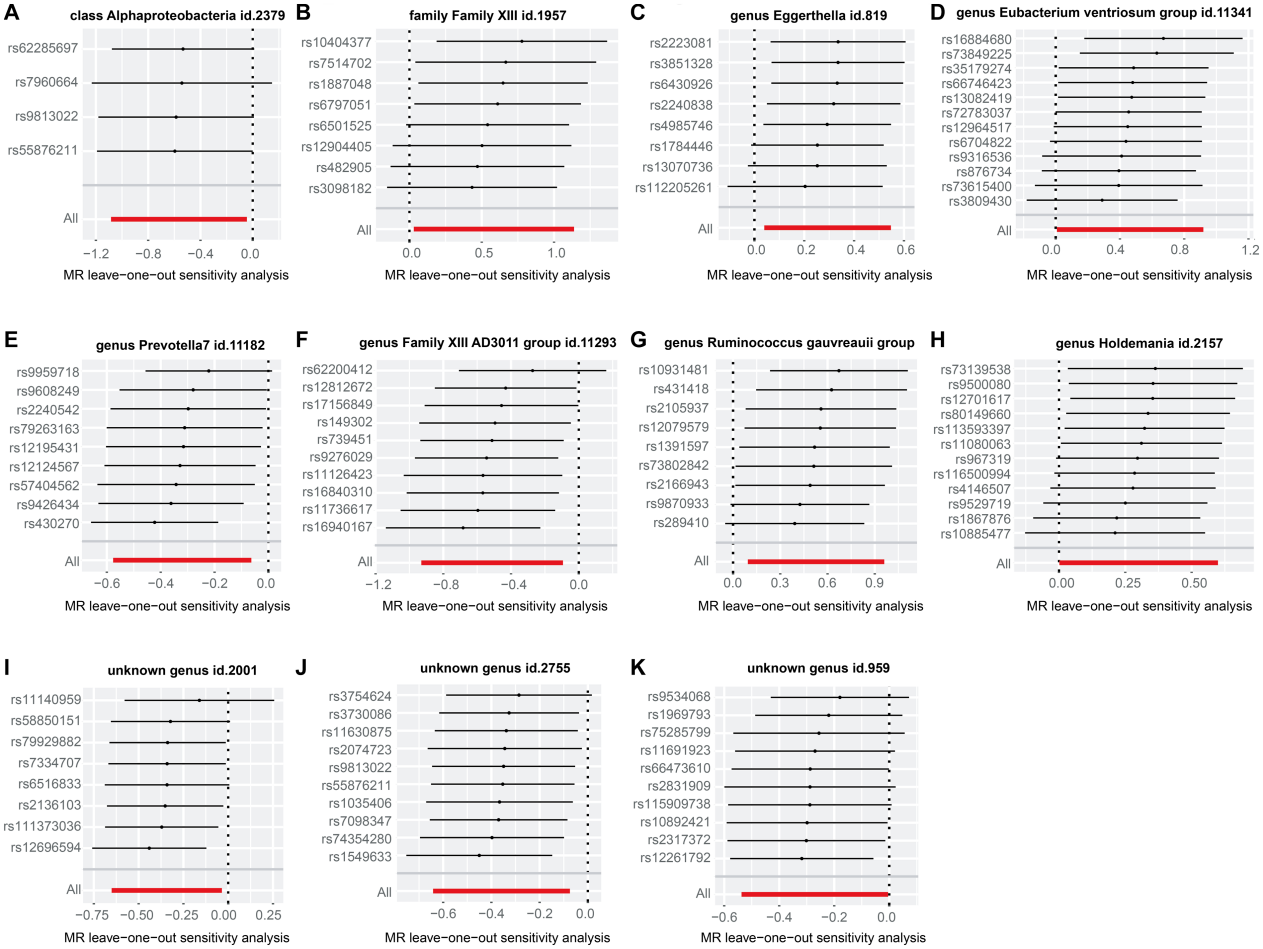
“Leave-one-out” plots for the causal association between genetically predicted gut microbiotas and CHB. These gut microbiotas included class *Alphaproteobacteria* **(A)**, family *Family XIII* **(B)**, genus *Eggerthella* **(C)**, genus *Eubacterium ventriosum* group **(D)**, genus *Prevotella 7* **(E)**, genus *Family XIII AD3011* group **(F)**, *Ruminococcus gauvreauii* group **(G)**, genus *Holdemania* **(H)**, unknown genus id.2001 **(I)**, unknown genus id.2755 **(J)**, and unknown genus id.959 **(K)**.

As shown in Supplementary Table S3, we noted a 0.35–8.32% contribution to the total variation (*R*^2^ values) of the 207 gut microbiotas, and the *F* values ranged from 10.81 to 160.49—excluding the possibility of weak genetic tool variables. Cochran’s IVW *Q* test revealed no significant heterogeneity among these IVs (Supplementary Table S4). Additionally, except for the genus *Eubacterium ventriosum* group (*p* = 0.034), MR-Egger regression intercept analysis showed no significant horizontal pleiotropy among the other 206 gut microbiotas (Supplementary Table S5). Further MR-PRESSO analysis did not reveal horizontal pleiotropy between the genus *Eubacterium ventriosum* group (Supplementary Table S6, global test *p*-value = 0.991) and CHB. Notably, in the resulting diagram of the leave-one-out method analysis, we observed abnormal IVs in family Family XIII, genus *Eubacterium ventriosum* group, genus *Holdemania*, and unknown genus id.959 (Figure 3). However, except for the genus *Ruminococcus gauvreauii* group, MR-PRESSO analysis did not reveal any significant outliers (global test *p* > 0.05, Supplementary Table S6). In addition, when rs431418 was excluded, outlier-corrected MR-PRESSO analysis did not disclose any horizontal pleiotropy in the genus *Ruminococcus gauvreauii* group (global test *p* = 0.122, Supplementary Table S6). Therefore, there was insufficient evidence to demonstrate horizontal pleiotropy between these gut microbiotas and CHB.

In reverse MR analysis, we identified 11 SNPs (*p* < 1 × 10^−5^; *R*^2^ < 0.001) significantly associated with CHB, explaining 45.5% of the total variation (summaries and details for each SNP are presented in Supplementary Table S7). The results of reverse MR analysis did not reveal any significant causal relationship between CHB and the 11 gut microbiotas mentioned above (Supplementary Table S8). Cochran’s Q statistics indicated no notable heterogeneity (Supplementary Table S9, *p* > 0.05), and the MR-Egger intercept test (Supplementary Table S10) and MR-PRESSO global test (Supplementary Table S11) suggested no evidence of pleiotropy.

The genetic correlation between each gut microbe and CHB is presented in Supplementary Table S12. The results suggest that there is a significant genetic correlation between gut microbiota such as genus *Family XIII AD3011*, genus *Holdemania*, class Alphaproteobacteria, genus *Eubacterium ventriosum*, genus *Eggerthella*, genus *Ruminococcus gauvreauii*, family Family XIII and chronic hepatitis B.

Finally, we employed ImageGP to illustrate the relative abundance of bacteria in individuals exhibiting a non-zero abundance of the 11 gut microbiota significantly associated with chronic hepatitis B, along with the average read count of these 11 gut microbiota across the study participants (see Supplementary Figure S1). Evidently, a substantial majority of these 11 gut microbiota are observed in over 50% of the population. The fecal transplantation of any of these 11 gut microbiota could thus ensure its applicability to a broad segment of the population.

## Discussion

Despite the significant progress made in reducing annual hepatitis B virus (HBV) infections through the use of vaccines and antiviral drugs, HBV remains a substantial economic burden and health threat to society (Lok et al., 2016; Tan et al., 2021). To effectively treat patients with chronic liver disease and gain further insights into its pathogenesis, it is crucial to explore appropriate interventions and treatment strategies.

The relationship between gut microbiota and CHB represents an intricate intersection of microbial ecology, host metabolism, and hepatic immunology. Emerging evidence points to alterations in the gut microbiome, known as dysbiosis, playing a significant role in the progression of CHB. The gut-liver axis serves as a bidirectional communication channel where microbial metabolites and pathogen-associated molecular patterns (PAMPs) can affect liver inflammation and fibrosis. In CHB patients, dysbiosis may lead to an increased permeability of the gut barrier, allowing the translocation of bacterial products into the liver and subsequently triggering inflammatory responses. This can contribute to liver damage and exacerbate the disease progression. Moreover, the complex interplay of gut microbiota with antiviral immune responses may influence the efficacy of antiviral treatments in CHB. Some researchers are investigating therapeutic interventions, such as the use of probiotics or prebiotics, to modulate the gut microbiome and potentially improve clinical outcomes for CHB patients. However, the mechanistic understanding of how gut microbiota influences CHB is still in its infancy, and further rigorous investigations are warranted to translate these findings into clinical practice (Chen et al., 2011; Lu et al., 2011; Wang et al., 2017).

In this study, we conducted the first-ever investigation into the causal relationship between gut microbiota and CHB using the summary statistics of the largest genome-wide meta-analysis of CHB and gut microbiotas conducted by the MiBioGen consortium. MR is less prone to confounding factors because germline genetic variation is randomly assigned during meiosis and therefore reflects exposure without being affected by reverse causality. The results of our bidirectional, two-sample MR study suggested that genetic predisposition to family Family XIII (OR = 1.79), genus *Eggerthella* (OR = 1.34), genus *Eubacterium ventriosum* group (OR = 1.59), genus *Holdemania* (OR = 1.35), and genus *Ruminococcus gauvreauii* group (OR = 1.69) was associated with a 79, 34, 59, 35, and 69% increased risk of CHB, respectively. Genetically predicted class Alphaproteobacteria (OR = 0.57), genus *Family XIII AD3011* group (OR = 0.60), and genus *Prevotella 7* (OR = 0.73) were also associated with a 43, 40, and 27% decreased risk of CHB, respectively. However, reverse MR analysis displayed no significant causal relationship between CHB and gut microbiota. In addition, various validation methods were used to ensure the reliability and robustness of our findings.

The genus *Ruminococcus gauvreauii* group belongs to the family Ruminococcaceae, which is an integral part of the Firmicutes phylum. Members of this genus are widely recognized as critical constituents of the gut microbiota in both humans and other animals (Chen et al., 2021). It has been hypothesized that these bacteria may influence liver inflammation and fibrosis through the modulation of short-chain fatty acids production, bile acid metabolism, and interaction with Toll-like receptors, thus impacting the overall gut-liver axis. Komiyama et al. (2021) and Shen et al. (2023) demonstrated that the intestinal microbial abundance of the genus *Ruminococcus gauvreauii* group increased significantly in patients with hepatitis B and C, and this bacterial group was also used as a biomarker of liver cancer caused by these two pathogens. This result was similar to our findings in that the *Ruminococcus gauvreauii* group increased the risk of CHB.

Several observational studies and a meta-analysis showed that genus *Eggerthella* was closely related to colitis, cancer, and mental health disorders (Nikolova et al., 2021; Alexander et al., 2022; Bai et al., 2022); and that it was able to induce Th17 activation by relieving the inhibition of Th17 transcription factor Ror γ t, a cell-and-antigen-independent mechanism (Alexander et al., 2022). Several mechanistic studies have shown that Th-17 cells and their secreted cytokine IL-17 promote the progression and long-term survival of CHB (Ge et al., 2010; Shen et al., 2023). In the current study, *Eggerthella* significantly increased the risk of CHB, most likely also through the Th-17 pathway.

The genus *Eubacterium ventriosum* group and genus *Holdemania* are both part of the Firmicutes phylum and are found within the human gut microbiome. The *Eubacterium ventriosum* group is recognized for its anaerobic properties and ability to ferment carbohydrates, while *Holdemania* is a less characterized genus known for its presence in various environments, including the gastrointestinal tract. In the context of hepatitis, particularly chronic liver diseases, these genera might be involved in modulating hepatic inflammation. Many observational studies have also revealed increased intestinal abundances of the genus *Eubacterium ventriosum* group and genus *Holdemania* in patients with various inflammatory diseases (Jang et al., 2020; Moreno-Arrones et al., 2020; Radjabzadeh et al., 2022; Ye et al., 2022). Alterations in their abundance could impact gut barrier integrity, allowing the translocation of microbial products into the liver, thereby triggering inflammatory responses. Though the exact mechanisms remain incompletely understood, some studies have indicated that shifts in the composition of gut microbiota, including these genera, might be correlated with liver disease progression and could influence the host’s immune responses to hepatic infections (Bajaj et al., 2014; Ren et al., 2019). These studies further confirmed our results of the *Eubacterium ventriosum* group and *Holdemania* as significantly increasing the risk of CHB.

The class Alphaproteobacteria includes Gram-negative, free-living, symbiotic and obligate intracellular bacteria and important plant, animal, and human pathogens (Hallez et al., 2017). Zhang et al. (2019) discerned that class Alphaproteobacteria was significantly lower in patients with chronic and acute liver failure associated with hepatitis B than in the control group. Similarly, we ascertained that class Alphaproteobacteria reflected a protective causal relationship with respect to CHB.

The genus *Prevotella 7*, part of the Bacteroidetes phylum, is a group of gram-negative bacteria that are widely recognized as essential constituents of the human gut microbiota. They are involved in the fermentation of polysaccharides, thereby contributing to producing short-chain fatty acids (SCFAs) such as butyrate, which plays a crucial role in maintaining gut barrier integrity and modulating the host’s immune response. In addition, it has been reported that *Prevotella 7* participates in various inflammatory pathologic processes by producing redox proteins or increasing host resistance (Hofer, 2014). Observational studies by Juan et al. also showed that *Prevotella 7* was associated with a reduced risk of gastric cancer (Wu et al., 2018). In the context of hepatitis, *Prevotella 7* might have protective effects. This protective effect is speculated to arise from its potential role in enhancing intestinal barrier function, which may limit the translocation of microbial metabolites and prevent their pro-inflammatory effects in the liver. Moreover, the immunomodulatory effect exerted by SCFAs can potentially attenuate hepatic inflammation, thereby preventing the progression of hepatitis (Shen et al., 2017). In our research, *Prevotella 7* was the protective factor against CHB and may thus reduce the risk of CHB by regulating the inflammatory and immune responses of the host. While promising, these observations require further investigation to fully elucidate the role of *Prevotella 7* in liver health.

The genus *Family XIII AD3011* group represents a less well-characterized classification within the Firmicutes phylum. Research by Lin et al. (2022) showed that the intestinal abundance of the genus *Family XIII AD3011* group in psoriatic arthritis was significantly lower than that in the normal group. However, there is a paucity of research on the relationship between the genus *Family XIII AD3011* group and CHB. Importantly, our study revealed for the first time that the genus *Family XIII AD3011* group exhibited a protective causal relationship concerning CHB.

### Strengths and limitations

This study possessed several advantages. Genetic variation in gut microbiota and CHB were obtained from the most extensive available GWAS meta-analysis of the MiBioGen consortium and GWAS catalog, ensuring the strength of the instruments applied in the MR analyses. The GWAS data we used encompassed many races worldwide, including those in Europe, Asia, and Australia, ensuring the universal applicability of our findings.

Some limitations to our investigation should also be acknowledged. First, the data from gut microbiota and CHB included both Europeans and Asians, where a population stratification bias may exist. However, at present, there is no GWAS summary data of large gut microbiota of the same race, and most of the GWAS data of CHB are from East Asian people (Trépo et al., 2014) only the GWAS summary data of CHB used in this study includes both European and East Asian races. Thus, although this partial bias was inevitable, we employed as many analytical methods as possible to eliminate this potential error. Second, there was no assessment of the existence of survival bias. In the future, separate MR analyses should be conducted for different age groups, and the consistency of the results should be assessed. The SNPs used in the analysis did not reach the traditional GWAS significance threshold (i.e., *p* < 5 × 10^−8^); however, we need to include additional genetic variation as IV for sensitivity analysis and the accurate detection of horizontal polymorphism. When the *p* value was set at 5 × 10^−8^ or 1 × 10^−6^, only one SNP or none were available for each microbe. For this, we used FDR correction to restrict the possibility of false positives. There are many high-level studies screened this way (Kurilshikov et al., 2021; Li et al., 2022; Long et al., 2023). Finally, the bacterial groups were analysed solely at the order or family level. Implementing additional advanced macro genome sequencing analysis in the future will thus enable the results to be more specific and accurate.

## Conclusion

Through this two-sample MR study, we established a causal link between gut microbiota and CHB, identifying specific gut microbiotas that either protect or exacerbate CHB risk. This information could potentially contribute to the clinical prevention and treatment of CHB through fecal microbiota transplantation.

## Data availability statement

The original contributions presented in the study are included in the article/Supplementary material, further inquiries can be directed to the corresponding author.

## Ethics statement

The data we used were from published studies approved by the appropriate ethics committees, and no further ethical approval was required for this study.

## Author contributions

QZ: conceptualization, methodology, software, investigation, visualization, and writing of the revised draft. XZ and RM: data provision and methodology. JZ: conceptualization, writing-revised draft, and funding acquisition. CZ: conceptualization, writing-original draft, funding acquisition, and project supervision. All authors contributed to the article and approved the submitted version.

## Conflict of interest

The authors declare that the research was conducted in the absence of any commercial or financial relationships that could be construed as a potential conflict of interest.

## Publisher’s note

All claims expressed in this article are solely those of the authors and do not necessarily represent those of their affiliated organizations, or those of the publisher, the editors and the reviewers. Any product that may be evaluated in this article, or claim that may be made by its manufacturer, is not guaranteed or endorsed by the publisher.

## Footnotes

^1^ https://sb452.shinyapps.io/power/

## Abbreviations

MR: Mendelian randomization
ML: Maximum likelihood
IVW: Inverse variance weighted
MR-PRESSO: MR Pleiotropy RESidual Sum and Outlier
GWAS: Genome-wide association study
CHB: Chronic hepatitis B
SNP: Single nucleotide polymorphism
