# Supplementary material for: Mendelian randomization supports causality between gut microbiota and chronic hepatitis B
Source: Front Microbiol. 2023 Aug 16;14:1243811. doi: 10.3389/fmicb.2023.1243811 (PMC10467284; doi:10.3389/fmicb.2023.1243811)
Supplement: Supplementary file 2 [file Image_1.pdf]

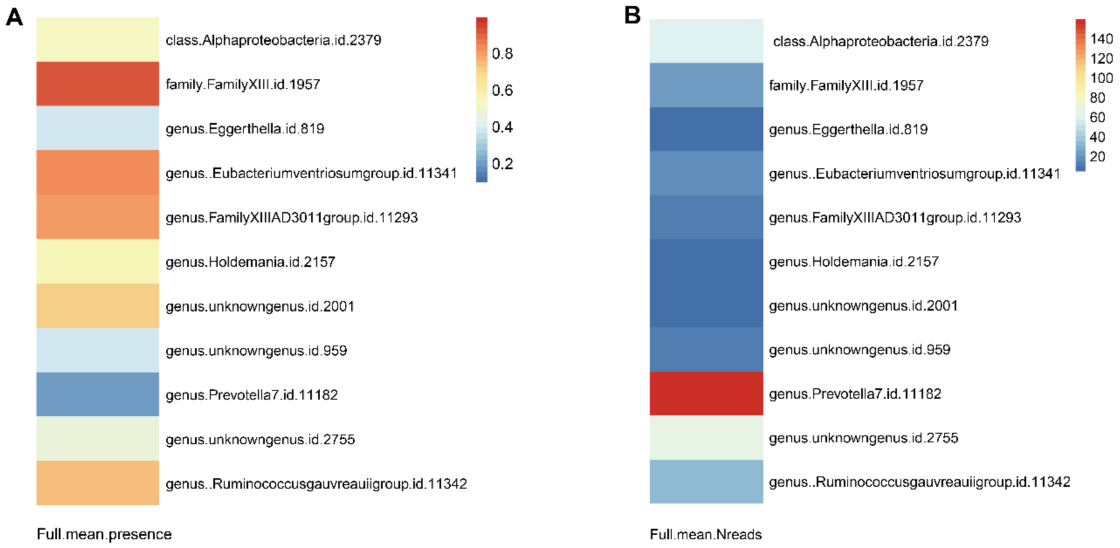

Supporting Figure 1. A.The Full.mean.presence of 11 gut microbiota significantly associated with chronic hepatitis B; B. The Full.mean.Nreads of 11 gut microbiota significantly associated with chronic hepatitis B; Full.mean.presence - the proportion of bacteria in individuals who have non-zero abundance of this bacteria. Full.mean.Nreads - the mean number of reads in non-zero participants (out of 10,000).
